# Effects of the timing of laparoscopic cholecystectomy after endoscopic retrograde cholangiopancreatography on liver, bile, and inflammatory indices and cholecysto-choledocholithiasis patient prognoses

**DOI:** 10.6061/clinics/2021/e2189

**Published:** 2021-03-30

**Authors:** Ming-Jun Gao, Zhi-Lan Jiang

**Affiliations:** I Department of Gastroenterology, Taizhou People’s Hospital, Taizhou City, Jiangsu Province, China; II Department of Immunization Program, Center for Disease Control and Prevention of Hailing District, Taizhou City, Jiangsu Province, China

**Keywords:** Bile Biochemistry, Choledocholithiasis, ERCP, LC, Inflammatory Reaction

## Abstract

**OBJECTIVES:**

This study explored the effects of the timing of laparoscopic cholecystectomy (LC) after endoscopic retrograde cholangiopancreatography (ERCP) on liver function, bile biochemical indices, inflammatory reactions, and cholecysto-choledocholithiasis patient prognoses.

**METHODS:**

A total of 103 cholecysto-choledocholithiasis patients were stratified into control (CG; n=51; LC at 4-7 d after ERCP) and observation groups (OG; n=52; LC at 1-3 d after ERCP) using a random number table.

**RESULTS:**

The surgical time was shorter and intraoperative blood loss was less in OG than in CG, and the two groups were not statistically different in terms of time to the first passage of gas through anus, length of postoperative hospital stay, conversion rate to laparotomy, and stone-free rate. Four weeks after LC, alanine aminotransferase (ALT), total bilirubin (TBil), albumin (ALB), and glutamyl transpeptidase (GGT) levels declined in both groups, but the difference was not statistically significant. Three days after LC, total bile acid (TBA) levels increased, and cholesterol (CHO), unconjugated bilirubin (UCB), and TBiL levels were reduced in both groups, but were not statistically different (*p*>0.05). Three days after LC, interleukin (IL)-6, procalcitonin (PCT), and high-sensitivity C-reactive protein (hs-CRP) levels in the serum and bile increased in both groups and were lower in OG. The total incidence of perioperative complications was 1.92% in OG, which was lower than 15.69% in the CG.

**CONCLUSION:**

For cholecysto-choledocholithiasis patients, LC at 1-3 d after ERCP can shorten surgical times, reduce intraoperative blood loss, improve liver function and bile biochemistry, relieve inflammatory reactions, reduce complications, and improve prognoses.

## INTRODUCTION

Cholecystolithiasis is a common disease with a case rate of approximately 7% in China. An estimated 10-18% of all patients with cholecystolithiasis present with choledocholithiasis as a complication (1). Cholecysto-choledocholithiasis is mainly treated surgically, including endoscopic retrograde cholangiopancreatography (ERCP) for pathological diagnosis and treatment of calculus of the bile duct, and laparoscopic cholecystectomy (LC), as a standard option for cholecystolithiasis treatment. The combination of ERCP and LC forms a minimally invasive therapy for cholecysto-choledocholithiasis and is commonly utilized in the clinical scenario (2). However, ERCP is associated with numerous complications, such as hemorrhage of the digestive tract, hyperamylasemia, and acute pancreatitis, which may affect the efficacy of LC (3,4). Therefore, the appropriate interval between ERCP and LC remains controversial. This study observed the effects of LC treatments 4-7 d and 1-3 d after ERCP on cholecysto-choledocholithiasis patients and evaluated patient improvements in liver function, bile biochemical indices, inflammatory reactions, and prognoses.

## MATERIALS AND METHODS

### General materials

A total of 103 cholecysto-choledocholithiasis patients admitted to our hospital between January 2017 and April 2019 were selected as observation subjects. Inclusion criteria were as follows: patients who were diagnosed with cholecysto-choledocholithiasis by abdominal B-ultrasound, computed tomography (CT), and magnetic resonance imaging cholangiopancreatography; those without lower common hepatic duct stenosis, bile duct variation, and Mirizzi syndrome; those who presented stone diameters ≤5 mm; those who met surgical indications; those without contraindications to ERCP and LC; those who underwent both ERCP and LC by the same group of surgeons; and those who provided informed consent to participate in the study. Exclusion criteria were as follows: subjects presenting malignant tumors, acute severe angiocholitis, infectious diseases, serious abdominal adhesions, multiple stones in the liver, failure of vital organs, acute pancreatitis, combined acute pancreatitis; patients with previous history of pancreatitis, multiple pancreatic duct angiography and other postoperative acute pancreatitis risk factors; and those with a history of biliary tract surgery. The subjects were stratified into control (CG; n=51) and observation groups (OG; n=52) using a random number table.

## METHODS

LC was performed after 4-7 d of ERCP in CG and after 1-3 d in OG.

### Endoscopic retrograde cholangiopancreatography

Patients were confirmed to have normal gastric and duodenal anatomical structures before surgery. ERCP was not suitable for patients whose anatomical structures had been damaged by previous cholangiojejunostomy, Billroth II subtotal gastrectomy, esophagojejunostomy, etc., and who manifested pancreatitis or suppurative cholangitis. Patients were locally anesthetized while lying prostrate. A duodenoscope was threaded through the mouth into the common bile duct, and 10 ml of 30% cardiografin (contrast medium) was injected. The anatomic structure of the common bile duct was observed, and stones were located, measured, and counted. An electric papillary knife was inserted into the common bile duct to produce an incision of approximately 10-15 mm in length in 12 o'clock point from the sphincter of Oddi. Alternatively, balloon dilation was used to dilate the sphincter of Oddi by 1 cm. Mechanical lithotripsy was applied, and stones were removed using a balloon catheter or stone basket. Next, the biliary tract was rinsed and imaged in case any residues were present. Nasobiliary drainage was performed. Patients had fasted for 1 d, were administered antibiotics to prevent infection, and subjected to vital sign monitoring for 24h. LC was performed in patients who had no discomfort, could ingest food normally, and passed examinations of urine, stool, blood, hemodiastase, and liver function.

### Laparoscopic cholecystectomy

LC was performed 4-7 d after ERCP in CG and 1-3 d in OG. Patients were administered general anesthesia while lying with the head positioned upright, the feet lowered, and the left side inclined by 30°. An incision of 10 mm was created along the inferior border of the navel to obtain a pneumoperitoneum pressure of 12 mmHg. A 10 mm Trocar was inserted into the incision to facilitate observation. Incisions (10 mm each) were also made below the cartilago ensiformis and right costal margin. A 10 mm Trocar was inserted into the incisions to serve as the surgical port. The surgeon explored the abdominal cavity, dissected and separated the Calot's triangle, pinched off the cystic gall duct with absorbable clips, and excised the cystic artery under the laparoscope. The middle part of the cystic duct was cut open, the contrast catheter was inserted into the common bile duct through the incision, and approximately 10 ml of 30% diatrizoate meglumine was injected to observe whether the common bile duct had a defect shadow. Next, the gallbladder bed, gallbladder, and serous membrane were separated, the gallbladder terminal was excised, and the bladder was ablated. An indwelling drainage tube was inserted into the abdominal cavity after cleaning and rinsing. All incisions were sutured.

Venous blood was collected at 6h and 24h after ERCP in both groups. Serum amylase was detected, and abdominal pain, fever, and other symptoms were observed.

### Evaluation criteria

#### General surgical indices

Surgical times, intraoperative blood loss, time to gastrointestinal motility, length of postoperative hospital stay, and stone-free rate were recorded.

#### Liver function

Before and 4 weeks after surgery, 5 ml blood samples were drawn and centrifuged at 2,500 rpm for 15 min. The liquid supernatant was collected and frozen for future quantification of ALT, TBil, ALB, and GGT levels using an automatic biochemistry analyzer.

#### Bile biochemical indices

During and 3 d after LC, 5 ml of bile was collected through the drainage tube at 8:00 a.m. to quantify levels of TBA, CHO, UCB, and TBil using the enzyme cycle method.

#### Serum inflammatory factors

Before and 3 d after LC, 3 ml of blood was drawn and centrifuged at 3,000 rpm for 10 min. The liquid supernatant was collected and frozen for future testing of IL-6 using an enzyme-linked immunosorbent assay, PCT using an enzyme-linked immunofluorescent assay (ELFIA), and hs-CRP using a turbidimetric inhibition immunoassay (TIIA).

#### Bile inflammatory factors

During and 3 d after LC, 3 ml of bile was drawn and centrifuged at 3,000 rpm for 10 min. The liquid supernatant was collected and frozen for future PCT testing using ELFIA and hs-CRP using TIIA.

#### Perioperative complications

Perioperative complications, including fervescence, bleeding, bile duct leaks, pancreatitis, elevated white blood cell counts, and hyperamylasemia were observed. The criteria for hyperamylasemia was the increased level of serum amylase 3h after surgery. Criteria for pancreatitis were as follows: (1) abdominal pain characteristic of acute pancreatitis, (2) serum amylase and/or lipase ≥3 times the upper limit of normal value, and (3) CT characteristic of acute pancreatitis. Serum amylase and lipase levels should be examined immediately when the patient has abdominal pain characteristic of acute pancreatitis. If serum amylase and/or lipase level is ≥3 times the upper limit of normal value, the diagnosis is confirmed. If serum amylase and/or lipase is <3 times the upper limit of normal value, CT examination was performed to assist diagnosis. If the patient did not have abdominal pain symptoms characteristic of acute pancreatitis, but serum amylase and/or lipase is ≥3 times the upper limit of normal value, acute pancreatitis was diagnosed.

#### Prognosis

Patients were followed up for 1 year to obtain the incidences of stone recurrence, angiocholitis, and traumatic bile duct stricture. According to the Bismuth classification of traumatic bile duct stricture are the following types: type I: the length of the common hepatic duct or bile duct stump below the confluence of the left and right hepatic ducts is ≥2 cm; type II: the length of the common hepatic duct stump below the confluence of the left and right hepatic ducts is <2 cm; type III: the confluence of the left and right hepatic ducts is complete, and the left and right hepatic ducts are connected; type IV: the confluence of the left and right hepatic ducts is damaged, and the left and right hepatic duct systems are isolated and disconnected; type V: type I, II, or III plus right accessory hepatic duct or vagal bile duct stricture.

### Statistical analysis

Statistical analysis was performed using SPSS software version 25.0 (IBM Corp., Armonk, NY, USA). In the case of numerical data expressed as the mean±standard deviation, comparison studies were performed using *t-*tests; in the case of nominal data expressed as a percentage, comparison studies were performed using the Chi-square test for intergroup comparisons. For all statistical comparisons, significance was defined as *p*<0.05.

### Ethics

This study was approved by the Ethics Committee of the Center for Disease Control and Prevention of Hailing District. All study participants provided written informed consent before participating in the study.

## RESULTS

### Intergroup comparison of general surgical indices

Gender, age, the diameter of the largest stone, the average stone count in the common bile duct, the diameter of the common bile duct, and the Child-Pugh grading of liver function were not statistically different, but comparable between the two groups (*p*>0.05) (Table 1). After surgery, the observation group had 25 cases of single stone and 27 cases of multiple stones in the common bile duct, while the control group had 23 cases of single stone and 29 cases of multiple stones in the common bile duct.

### Intergroup comparison of surgical time and length of hospital stay

The surgical time was shorter and intraoperative blood loss was less in OG than in CG (*p*<0.05), but the difference in the length of postoperative hospital stay was not significant (*p*>0.05), suggesting that the performance of LC 1-3 d after ERCP could significantly shorten surgical times and reduce stress-induced injuries due to surgical treatment of cholecysto-choledocholithiasis patients (Table 2).

### Intergroup comparison of general surgical indices

The time to the first passage of gas through the anus and the stone-free rate were compared between the two groups (*p*>0.05). These findings suggest that LC after ERCP at different surgical times had minimal effect on the recovery of gastrointestinal function and stone-free rates of cholecysto-choledocholithiasis patients (Table 3).

### Intergroup comparison of liver function

**Figure 1:**
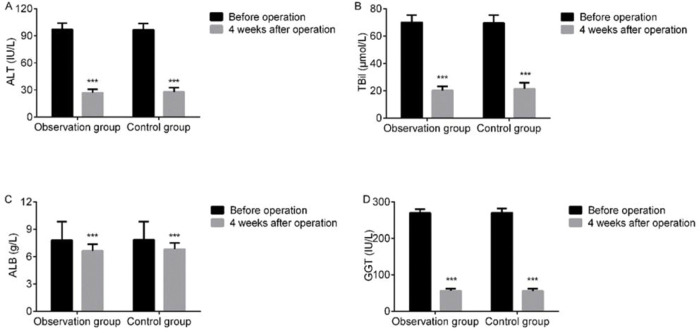
Intergroup comparison of liver function. At 4 weeks after LC, serum ALT, TBil, ALB, and GGT levels declined in both groups but were not statistically different between the two groups (*p*>0.05). A: ALT; B: TBil; C: ALB; D: GGT. Note: ***p<0.001 *versus* preoperative values.

At 4 weeks after LC, serum ALT, TBil, ALB, and GGT levels declined in both groups, but were not statistically different between the two groups (*p*>0.05). This finding indicated that LC after ERCP at different surgical times had a limited effect on the liver function of cholecysto-choledocholithiasis patients (Figure 1).

### Intergroup comparison of biochemical bile indices

**Figure 2:**
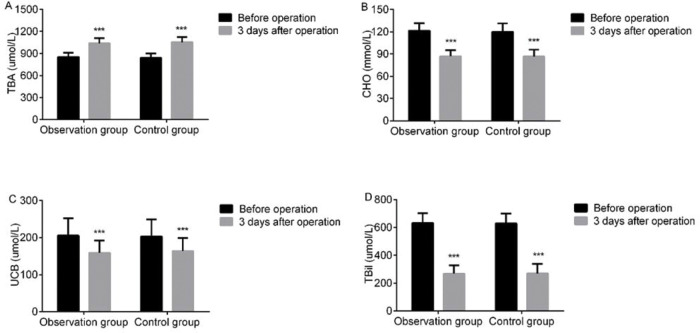
Intergroup comparison of bile biochemical indices. At 3 d after LC, bile TBA levels were elevated, while CHO, UCB, and TBiL levels declined in both groups, but the intergroup difference was not statistically significant (*p*>0.05). A: TBA; B: CHO; C: UCB; D: TBiL. Note: ***p<0.001 *versus* preoperative values.

At 3 d after LC, bile TBA levels were elevated, while CHO, UCB, and TBiL levels declined in both groups, but the intergroup difference was not statistically significant (*p*>0.05). These findings suggest that LC after ERCP at different surgical times had limited impact on the biochemical bile indices of cholecysto-choledocholithiasis patients (Figure 2).

### Intergroup comparison of serum inflammatory reactions

**Figure 3:**
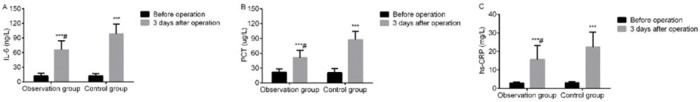
Intergroup comparison of serum inflammatory reactivity. At 3 d after LC, the serum IL-6, PCT, and hs-CRP levels increased in both groups but were lower in OG than in CG (*p*<0.05) A: IL-6; B: PCT; C: hs-CRP. Note: ***p<0.05 *versus* preoperative values.

At 3 d after LC, the serum IL-6, PCT, and hs-CRP levels increased in both groups, but were lower in OG than in CG (*p*<0.05), suggesting that LC 1-3 d after ERCP could significantly reduce serum inflammatory reactions in cholecysto-choledocholithiasis patients (Table 4, Figure 3).

### Intergroup comparison of bile inflammatory reactions

**Figure 4:**
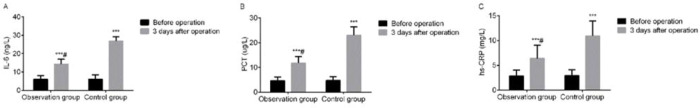
Intergroup comparison of bile inflammatory reactivity. At 3 d after LC, bile IL-6, PCT, and hs-CRP levels increased in both groups but were lower in OG than in CG (*p*<0.05). A: IL-6; B: PCT; C: hs-CRP. Note: ***p<0.05 *versus* preoperative values.

At 3 d after LC, bile IL-6, PCT, and hs-CRP levels increased in both groups, but were lower in OG than in CG (*p*<0.05). These findings suggest that LC at 1-3 d after ERCP could significantly reduce inflammatory bile reactions in cholecysto-choledocholithiasis patients (Table 5, Figure 4).

### Intergroup comparison of perioperative complications

The total incidence of perioperative complications was 7.69% in OG and 29.41% in CG (*p*<0.05), indicating that LC at 1-3 d after ERCP can reduce perioperative complications in cholecysto-choledocholithiasis patients (Table 6).

### Intergroup comparison of prognosis

After 1-year of follow-up, the stone recurrence rate was compared between the two groups (*p*>0.05). The incidences of angiocholitis and bile duct stricture were lower in OG than in CG (*p*<0.05), suggesting that LC 1-3 d after ERCP could noticeably reduce the incidences of angiocholitis and bile duct stricture in cholecysto-choledocholithiasis patients (Table 7).

## DISCUSSION

Cholecysto-choledocholithiasis may cause complications such as acute or chronic angiocholitis, obstructive jaundice, biliary pancreatitis, and liver function injury, or could be even fatal (5). Therefore, patients diagnosed with cholecysto-choledocholithiasis should receive treatment immediately. Cholecystectomy with choledocholithotomy and T-tube drainage is a traditional invasive therapy, following which patients require time to recover (6). ERCP and LC comprise a minimally invasive treatment for cholecysto-choledocholithiasis, and it is extensively utilized in the clinical scenario.

According to some studies, the sphincter of Oddi could be damaged during ERCP, leading to bacterial colonization in the bile ducts, a cause of ligamentum hepatoduodenale infection. Furthermore, this damage may increase the difficulty of dissecting the Calot's triangle in LC and risk conversion to laparotomy accordingly (7-9). In the Netherlands, 45% of the 45 hospitals that require LC after ERCP and endoscopic sphincterotomy (EST) define the surgical time of LC at 6-12 weeks after ERCP+EST, and only four utilize an earlier date (10). At 6-12 weeks after ERCP+EST, inflammation was resolved, and LC complications were reduced. However, patients may experience repeated hospitalizations and increased medical expenses. In addition, the long-term delays after ERCP+EST may increase the risk of gallstones migrating into the common bile duct, as well as cholecystitis recurrence (11,12). This study compared the results of LC 4-7 d after ERCP and LC 1-3 d after ERCP. The two therapeutic regimens did not statistically differ in terms of time to the first passage of gas through the anus, the length of postoperative hospital stay, and the stone-free rates, but the average surgical time was shorter and intraoperative blood loss was less in LC 1-3 d after ERCP. A possible reason for this finding is that edema in the Calot's triangle occurred after ERCP due to the surgical wound, which mainly occurred 72h after surgery and worsened continuously, thus increasing the difficulty of LC 4-7 d after ERCP, resulting in prolonged surgical time and increased blood loss (13,14). According to the results of this study, the liver functions and bile biochemistry of both OG and CG improved, but these changes were not statistically significant. This finding suggested that LC after ERCP at different times can improve the liver function and bile biochemistry of cholecysto-choledocholithiasis patients. This may be because cholecysto-choledocholithiasis leads to obstruction of the bile duct. Consequently, bile cannot be discharged into the intestinal tract normally, thus increasing the discharge of combined bilirubin and TBA into the blood. Meanwhile, the amount of TBA in the blood increases, which induces oxidative stress and apoptosis of tissue cells, causing liver injury and increased bilirubin levels (15). ERCP+LC can solve the problem of stones obstructing the bile duct and cholestasis, reduce bile duct pressure and TBA loss due to bile penetration into the blood, improve the capacity of the TBA pool and bile components, promote the balance of bile acid, cholesterol, and phosphatide in bile, and reduce the injuries of the liver parenchyma and extrahepatic tissues caused by cholestasis, thereby improving liver function (16).

The increased levels of inflammatory factors, including IL-6, PCT, and hs-CRP, may occur in response to the surgical wound, which could be viewed as a measure of the degree of overall inflammatory reactivity. IL-6 is produced by T lymphocytes. IL-6 may increase due to injury or inflammatory reactions, and in turn, stimulate and activate T leukomonocytes (17). In healthy tissue, PCT is insignificantly expressed, but its expression increases sharply in the case of injury or infection (18). hs-CRP level, an important indicator for evaluating postoperative efficacy, increases rapidly following injury or as a consequence of autoimmune disease. Based on the results of this study, 3 d after LC, the levels of IL-6, PCT, and hs-CRP increased in the serum and bile of both groups, but were lower in OG, which is consistent with the findings of previous relevant studies (19). This finding suggests that the inflammatory reactions related to LC 1-3 d after ERCP are reduced. A possible reason for this finding is that 1-3 d after ERCP, edema is not apparent, which shortens LC surgical time and reduces complications, surgical injuries, and inflammatory stimulation of the abdomen and biliary tract.

Some researchers have asserted that LC within 7 d after ERCP could eliminate 76% of recurrent biliary complications (20-22). The comparative results of this study showed that LC at 1-3 d after ERCP was associated with fewer perioperative complications, lower long-term incidences of angiocholitis and bile duct stricture, and better prognoses, which are consistent with the results of a previous study (12). These improvements were attributed to the following reasons. Edema occurring within 72h after ERCP increased the difficulty of Calot's triangle dissection, conversion rate to LC at 4-7 d after ERCP, and incidence of complications. Meanwhile, increased difficulty of dissection leads to prolonged surgical durations. Both LC and laparotomy are invasive to the Calot's triangle and surrounding tissues and increase the long-term incidence of biliary tract complications. However, complications related to ERCP, including hemorrhage of the digestive tract, hyperamylasemia, and acute pancreatitis, develop within 12h after surgery (23). Therefore, LC 1-3 d after ERCP is safe and guarantees sufficient time to observe and remedy any complications related to ERCP. Thus, this study suggests that LC 1-3 d after ERCP is the most appropriate therapeutic strategy. However, some studies (24) believe that LC at the early or delayed stage of ERCP has no advantages in terms of stone clearance rate and complications, which may be caused by factors such as differences in included cases and the nature of the disease. Considering that there are many complications associated with ERCP, and serious complications can cause great harm to patients, and can even cause death, the application of ERCP should be avoided clinically, and non-invasive or minimally invasive methods should be adopted instead. For cholecysto-choledocholithiasis patients who need ERCP, such as those who cannot be diagnosed by B-ultrasound, whose lesions are close to the duodenal wall, whose bile duct is not dilated, and who is not suitable for laparotomy, etc., the treatment plan for LC within 1-3 d after ERCP should be considered. In addition, for patients with calculus migration to the main biliary tract requiring ERCP, treatment and observation can be conducted first, and after the improvement of symptoms and biliary function examination, LC can be performed, and intraoperative cholangiography can be performed. If choledocholithiasis is observed, ERCP can be performed intraoperatively to clear the main bile duct.

In conclusion, for cholecysto-choledocholithiasis patients, LC at 1-3 d after ERCP can shorten surgical duration, improve liver function and bile biochemistry, relieve inflammatory reactions, reduce complications, and improve overall prognoses. This therapeutic regimen deserves further consideration as a standard course of therapy. However, this study has few limitations. The number of subjects enrolled in this study was limited, and the samples were single. Further studies with broader sample data support are required.

## AUTHOR CONTRIBUTIONS

Gao MJ was responsible for the study design/planning, data interpretation and literarture analysis/search. Jiang ZL was responsible for the study design/planning, data collection/entry, data analysis/statistics, and manuscript preparation.

## Figures and Tables

**Table 1:** Intergroup comparison of general materials (x¯±s, *n*).

							Child-Pugh grading of liver function	
Group	*n*	Gender (M/F)	Mean age (y)	Diameter of the largest stone in common bile duct (cm)	Mean stone count in common bile duct (No.)	Diameter of common bile duct (cm)	A	B
OG	52	32/20	56.16±9.26	0.62±0.25	1.70±0.35	1.25±0.42	46	6
CG	51	29/22	57.39±8.28	0.59±0.22	1.68±0.31	1.29±0.45	44	7
χ2/t		0.233	0.710	0.646	0.307	0.467	0.112	
*p*		0.629	0.479	0.520	0.760	0.642	0.738	

**Table 2:** Intergroup comparison of surgical times and length of postoperative hospital stays [x¯±s, *n* (%)].

Group	*n*	Operation time (min)	Postoperative length of hospital stay (d)
OG	52	50.03±10.65	7.18±1.23
CG	51	69.79±15.31	7.25±1.26
χ2/t		7.616	0.285
*p*		0.000	0.776

**Table 3:** Intergroup comparison of general surgical indices [x¯±s, *n* (%)].

Group	*n*	Intraoperative blood loss (ml)	Time to the first passage of gas through anus (d)	Conversion rate to laparotomy (%)	Stone-free rate (%)
OG	52	109.62±25.73	2.06±0.33	1 (1.92)	49 (94.23)
CG	51	221.59±26.55	2.12±0.37	3 (5.88)	45 (88.23)
*χ^2^*/*t*		21.736	0.869	0.281	0.531
*p*		0.000	0.387	0.298	0.281

**Table 4:** Comparison of serum inflammatory reactivity between the two groups.

		IL-6 (ng/L)		PCT (μg/L)		hs-CRP (mg/L)	
Group	*n*	Before surgery	At 3 d after LC	Before surgery	At 3 d after LC	Before surgery	At 3 d after LC
OG	52	12.05±5.23	65.46±18.72*	21.49±7.13	50.94±15.13*	2.96±0.53	15.60±7.56*
CG	51	11.96±4.97	98.37±20.15*	20.52±8.19	87.25±17.22*	3.05±0.61	22.39±8.01*
*t*		0.090	8.590	0.642	11.374	0.799	4.425
*p*		0.929	0.000	0.523	0.000	0.846	0.000

* Note: compared with before surgery, *p*<0.05.

**Table 5:** Comparison of bile inflammatory reactivity between the two groups.

		IL-6 (ng/L)		PCT (μg/L)		hs-CRP (mg/L)	
Group	*n*	Before surgery	At 3 d after LC	Before surgery	At 3 d after LC	Before surgery	At 3 d after LC
OG	52	6.03±2.16	14.26±2.70*	4.65±1.47	11.79±2.66*	2.87±1.15	6.38±2.69*
CG	51	6.11±2.28	26.87±2.25*	4.73±1.52	23.05±3.32*	2.90±1.19	10.93±3.01*
*t*		0.183	25.724	0.272	19.014	0.130	8.093
*p*		0.855	0.000	0.787	0.000	0.897	0.000

* Note: compared with before surgery, *p*<0.05.

**Table 6:** Intergroup comparison of perioperative complications [*n* (%)].

Group	*n*	Fervescence	Bleeding	Leak in bile duct	Pancreatitis	High white blood cell count	Hyperamylasemia	Total incidence
OG	52	0 (0.00)	0 (0.00)	0 (0.00)	0 (0.00)	1 (1.92)	0 (0.00)	1 (1.92)
CG	51	2 (3.92)	1 (1.96)	1 (1.96)	1 (1.96)	2 (3.92)	1 (1.96)	8 (15.69)
*χ^2^*								4.512
*p*								0.013

**Table 7:** Intergroup comparison of prognoses [*n* (%)].

					Traumatic biliary stricture			
Group	*n*	Stone recurrence	Angiocholitis	Bile duct stricture	Grade I	Grade II	Grade III	Total
OG	52	1 (1.92)	0 (0.00)	0 (0.00)	0 (0.00)	0 (0.00)	0 (0.00)	0.00
CG	51	3 (5.88)	7 (13.72)	9 (17.65)	3 (5.88)	5 (9.80)	1 (1.96)	9 (17.65)
*χ^2^*		0.281	5.644	7.964				7.964
*p*		0.298	0.006	0.002				0.002
